# Differences in the prospective association between individual plasma phospholipid saturated fatty acids and incident type 2 diabetes: the EPIC-InterAct case-cohort study

**DOI:** 10.1016/S2213-8587(14)70146-9

**Published:** 2014-10

**Authors:** Nita G Forouhi, Albert Koulman, Stephen J Sharp, Fumiaki Imamura, Janine Kröger, Matthias B Schulze, Francesca L Crowe, José María Huerta, Marcela Guevara, Joline WJ Beulens, Geertruida J van Woudenbergh, Laura Wang, Keith Summerhill, Julian L Griffin, Edith JM Feskens, Pilar Amiano, Heiner Boeing, Françoise Clavel-Chapelon, Laureen Dartois, Guy Fagherazzi, Paul W Franks, Carlos Gonzalez, Marianne Uhre Jakobsen, Rudolf Kaaks, Timothy J Key, Kay-Tee Khaw, Tilman Kühn, Amalia Mattiello, Peter M Nilsson, Kim Overvad, Valeria Pala, Domenico Palli, J Ramón Quirós, Olov Rolandsson, Nina Roswall, Carlotta Sacerdote, María-José Sánchez, Nadia Slimani, Annemieke MW Spijkerman, Anne Tjonneland, Maria-José Tormo, Rosario Tumino, Daphne L van der A, Yvonne T van der Schouw, Claudia Langenberg, Elio Riboli, Nicholas J Wareham

**Affiliations:** a MRC Epidemiology Unit, University of Cambridge, Cambridge, UK; b MRC Human Nutrition Research, Cambridge, UK; c German Institute of Human Nutrition Potsdam-Rehbruecke, Potsdam, Germany; d Nuffield Department of Medicine, University of Oxford, Oxford, UK; e Department of Epidemiology, Murcia Regional Health Council, Murcia, Spain; f CIBER Epidemiología y Salud Pública (CIBERESP), Murcia, Spain; g Navarre Public Health Institute (ISPN), Pamplona, Spain; h University Medical Center Utrecht, Utrecht, Netherlands; i Wageningen University, Wageningen, Netherlands; j Public Health Division of Gipuzkoa, San Sebastian, Spain; k Instituto BIO-Donostia, Basque Government, San Sebastian, Spain; l Inserm, CESP, U1018, Villejuif, France; m Univ Paris-Sud, UMRS 1018, Villejuif, France; n Gustave Roussy Institute, F-94800 Villejuif, France; o Lund University, Malmö, Sweden; p Umeå University, Umeå, Sweden; q Catalan Institute of Oncology (ICO), Barcelona, Spain; r Department of Public Health, Section for Epidemiology, Aarhus University, Aarhus, Denmark; s German Cancer Research Centre (DKFZ), Heidelberg, Germany; t Department of Public Health and Primary Care, University of Cambridge, Cambridge, UK; u Dipartimento di Medicina Clinica e Chirurgia, Federico II University, Naples, Italy; v Aalborg University Hospital, Aalborg, Denmark; w Epidemiology and Prevention Unit, Fondazione IRCCS Istituto Nazionale dei Tumori, Milan, Italy; x Cancer Research and Prevention Institute (ISPO), Florence, Italy; y Public Health Directorate, Asturias, Spain; z Danish Cancer Society, Copenhagen, Denmark; aa Unit of Cancer Epidemiology, Citta' della Salute e della Scienza Hospital—University of Turin and Centre for Cancer Prevention (CPO), Turin, Italy; ab Human Genetics Foundation (HuGeF), Turin, Italy; ac Andalusian School of Public Health, Granada, Spain; ad Instituto de Investigación Biosanitaria de Granada (Granada.ibs), Granada, Spain; ae International Agency for Research on Cancer, Lyon, France; af National Institute for Public Health and the Environment (RIVM), Bilthoven, Netherlands; ag Danish Cancer Society Research Center, Copenhagen, Denmark; ah Department of Health and Social Sciences, Universidad de Murcia, Spain; ai Associazione Italiana Registri Tumori, Dipartimento di Prevenzione Medica, Azienda Sanitaria Provinciale, Ragusa, Italy; aj Aire Onlus, Ragusa, Italy; ak School of Public Health, Imperial College London, London, UK

## Abstract

**Background:**

Conflicting evidence exists regarding the association between saturated fatty acids (SFAs) and type 2 diabetes. In this longitudinal case-cohort study, we aimed to investigate the prospective associations between objectively measured individual plasma phospholipid SFAs and incident type 2 diabetes in EPIC-InterAct participants.

**Methods:**

The EPIC-InterAct case-cohort study includes 12 403 people with incident type 2 diabetes and a representative subcohort of 16 154 individuals who were selected from a cohort of 340 234 European participants with 3·99 million person-years of follow-up (the EPIC study). Incident type 2 diabetes was ascertained until Dec 31, 2007, by a review of several sources of evidence. Gas chromatography was used to measure the distribution of fatty acids in plasma phospholipids (mol%); samples from people with type 2 diabetes and subcohort participants were processed in a random order by centre, and laboratory staff were masked to participant characteristics. We estimated country-specific hazard ratios (HRs) for associations per SD of each SFA with incident type 2 diabetes using Prentice-weighted Cox regression, which is weighted for case-cohort sampling, and pooled our findings using random-effects meta-analysis.

**Findings:**

SFAs accounted for 46% of total plasma phospholipid fatty acids. In adjusted analyses, different individual SFAs were associated with incident type 2 diabetes in opposing directions. Even-chain SFAs that were measured (14:0 [myristic acid], 16:0 [palmitic acid], and 18:0 [stearic acid]) were positively associated with incident type 2 diabetes (HR [95% CI] per SD difference: myristic acid 1·15 [95% CI 1·09–1·22], palmitic acid 1·26 [1·15–1·37], and stearic acid 1·06 [1·00–1·13]). By contrast, measured odd-chain SFAs (15:0 [pentadecanoic acid] and 17:0 [heptadecanoic acid]) were inversely associated with incident type 2 diabetes (HR [95% CI] per 1 SD difference: 0·79 [0·73–0·85] for pentadecanoic acid and 0·67 [0·63–0·71] for heptadecanoic acid), as were measured longer-chain SFAs (20:0 [arachidic acid], 22:0 [behenic acid], 23:0 [tricosanoic acid], and 24:0 [lignoceric acid]), with HRs ranging from 0·72 to 0·81 (95% CIs ranging between 0·61 and 0·92). Our findings were robust to a range of sensitivity analyses.

**Interpretation:**

Different individual plasma phospholipid SFAs were associated with incident type 2 diabetes in opposite directions, which suggests that SFAs are not homogeneous in their effects. Our findings emphasise the importance of the recognition of subtypes of these fatty acids. An improved understanding of differences in sources of individual SFAs from dietary intake versus endogenous metabolism is needed.

**Funding:**

EU FP6 programme, Medical Research Council Epidemiology Unit, Medical Research Council Human Nutrition Research, and Cambridge Lipidomics Biomarker Research Initiative.

## Introduction

Saturated fatty acids (SFAs) are generally thought to have detrimental effects on health, as represented by the widespread public health message advising a reduction in SFA intake to less than 10% or even 7% of total energy to benefit cardiometabolic health, including lowering of type 2 diabetes risk.^1^ However, little evidence exists to support adverse effects of high SFA intake on risk of type 2 diabetes.^2^ Indeed, the Women's Health Initiative Diet Modification Trial^3^ suggested no benefit of a reduction in SFA intake on the incidence of type 2 diabetes. Accumulating evidence suggests that intake of dairy products, which are typically high in SFA content, is inversely associated with type 2 diabetes,^4^, ^5^ which, together with the null or inconsistent evidence about total SFA intake and risk of type 2 diabetes, has raised doubts about whether all SFA intake has adverse health effects.

Previous studies of dietary SFA intake have had inconclusive results, limited by measurement error of dietary assessment, and have focused on total SFA intake without analysis of SFA intake varying by carbon chain lengths. The objective measurement of SFAs with different carbon chain lengths in blood fractions enables assessment of individual SFAs.^6^ SFAs in blood can be directly interpreted as dietary SFAs for fatty acids that are good biomarkers of intake, such as 15:0 (pentadecanoic acid) and 17:0 (heptadecanoic acid), which are exogenously derived from dietary dairy fats.^6^, ^7^, ^8^ However, interpretation is more complex for SFAs like palmitic acid (16:0) and stearic acid (18:0), which are synthesised endogenously through de-novo lipogenesis stimulated by increased intake of carbohydrates and alcohol,^6^, ^9^, ^10^, ^11^, ^12^ and which might only partly represent dietary intake.^6^, ^13^ The extent to which different dietary components can induce de-novo lipogenesis varies.^11^ Additionally, uncertainties remain about the extent to which dietary SFAs are incorporated into blood SFAs, and the relative contribution of de-novo lipogenesis versus habitual diets to the amounts of SFAs circulating in the blood.^14^ However, the varying effects of different blood SFAs on the risk of type 2 diabetes are of scientific and public health interest. Only a few small studies have assessed a range of circulating SFAs,^15^, ^16^, ^17^, ^18^, ^19^, ^20^, ^21^ and evidence supporting associations of different blood SFAs with the incidence of type 2 diabetes is scarce.

In this large longitudinal study of the European Prospective Investigation into Cancer and Nutrition Study (EPIC)-InterAct study,^22^ we aimed to investigate the prospective associations between objectively measured individual SFAs in the plasma phospholipid fraction and incident type 2 diabetes. We also investigated associations of food consumption with circulating SFAs, and studied SFA metabolism indirectly by analysing ratios of relevant fatty acids.

## Methods

### Study design and population

**Figure 1:**
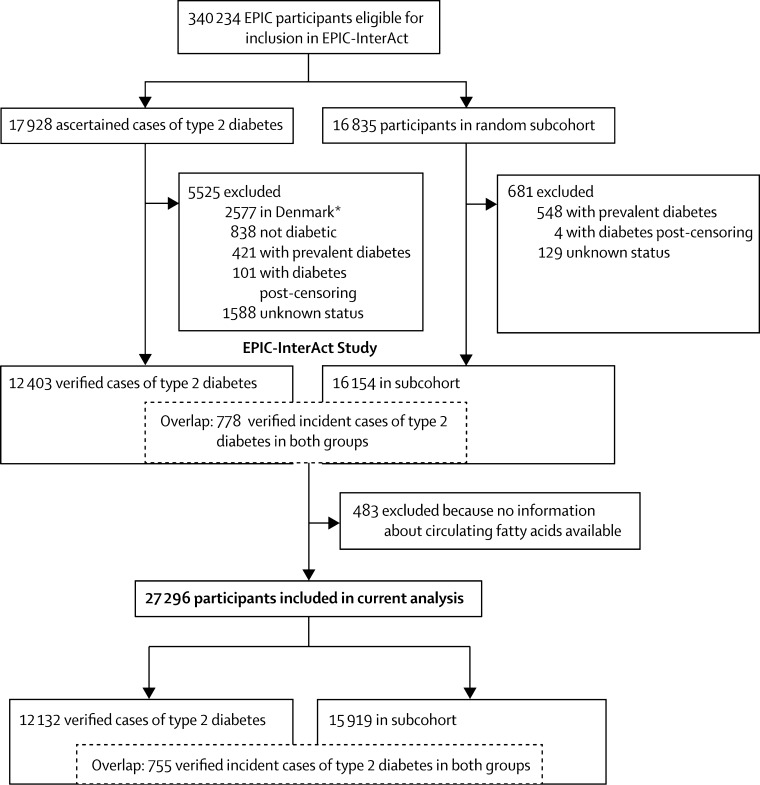
Case-cohort design of the InterAct study and the number of participants included in this analysis * A random sample of 2055 type 2 diabetes cases from Denmark was included after the exclusion of 2577 cases.

The methods of the InterAct project have previously been described in detail.^22^ To summarise, we did a case-cohort study that combines the temporal sequence and power advantages of a large prospective cohort with the measurement efficiency of a case-control study. Additionally, since the random subcohort is selected from the entire cohort independent of the outcome, it can be used as a comparison cohort for various outcomes of interest. From 340 234 people with 3·99 million person-years of follow-up (1991–2007) in eight countries of the EPIC study (France, Italy, Spain, UK, Netherlands, Germany, Sweden, and Denmark), we verified 12 403 cases of type 2 diabetes. From the EPIC cohort, we also used a random number generator to randomly select 16 835 people with baseline plasma samples in a subcohort. After exclusions for prevalent diabetes and uncertain diabetes status, 16 154 individuals remained in the subcohort, including 778 with incident type 2 diabetes during follow-up (a feature of the case-cohort design).^22^ From this case cohort of 27 779 participants, we excluded 483 for whom no fatty acid data were available, leaving 27 296 adults, among whom there were 12 132 cases of type 2 diabetes and 15 919 subcohort participants (including 755 incident cases of type 2 diabetes within the subcohort; figure 1). All participants provided written informed consent and the study was approved by all local ethics committees.

### Procedures

Incident type 2 diabetes was ascertained up until Dec 31, 2007, through a review of several sources of evidence, reported previously:^22^ self-report, linkage to primary care registers, secondary care registers, medication use (drug registers), hospital admissions, and mortality data. No diabetes cases were ascertained solely by self-report and we sought further evidence for all cases with information about incident type 2 diabetes from fewer than two independent sources at a minimum, including a review of individual medical records in some centres. Cases in Denmark and Sweden were identified from local and national diabetes and pharmaceutical registers and were judged to be verified.

Fatty acids were profiled at the Medical Research Council Human Nutrition Research (Cambridge, UK); profiling involved analysis of plasma samples stored at baseline at −196°C (or −150°C in Denmark)—a temperature at which fatty acids remain stable.^6^ The assay methods have previously been described^23^ and included hydrolysis and methylation to convert phospholipid fatty acids into more volatile fatty acid methyl esters and separation of the different fatty acids by gas chromatography (J&W HP-88, 30 m length, 0·25 mm internal diameter [Agilent Technologies, CA, USA]) equipped with flame ionisation detection (7890N GC [Agilent Technologies]). Samples from people with type 2 diabetes and subcohort participants were processed in random order by centre, and laboratory staff were masked to all participant characteristics by the use of anonymised aliquots.

We identified 37 different fatty acids by their retention times compared with those of commercial standards and expressed each level as percentage of total phospholipid fatty acids (mol%). These fatty acids included nine SFAs with relative concentrations higher than 0·05%: myristic acid (14:0), coefficient of variation 9·4%; pentadecanoic acid (15:0), 11·9%; palmitic acid (16:0), 1·6%; heptadecanoic acid (17:0), 4·2%; stearic acid (18:0), 2·0%; arachidic acid (20:0), 15·3%; behenic acid (22:0), 10·3%; tricosanoic acid (23:0), 18·9%; and lignoceric acid (24:0), 14·7%. We used human and equine plasma (Sera Laboratories International, West Sussex, UK) for quality control.^23^

Weight and height were measured by trained professionals using standardised protocols and were used to calculate BMI (in kg/m^2^). Waist circumference was measured by staff, with the exception of French participants and a subset of participants from Oxford, UK, who self-reported their measurements, and in Umeå, Sweden, where this parameter was not recorded. We used lifestyle questionnaires to assess demographics, smoking status, medical history, and education level. Physical activity was assessed by a validated questionnaire from which a four-point ordinal category of activity was derived. We assessed habitual diet during the past 12 months at baseline using country-specific validated food frequency questionnaires or diet histories. Total energy and nutrient intakes were based on the standardised EPIC Nutrient Database.^24^ HbA1_C_ was measured in the erythrocyte fraction from samples stored at −196°C using the Tosoh-G8 HPLC analyser (Tosoh Bioscience, Japan) at Stichting Huisartsen Laboratorium (Etten-Leur, Netherlands).

### Statistical analysis

We analysed the distribution of individual plasma phospholipid fatty acids and expressed them as mol%. We estimated country-specific hazard ratios (HRs) and 95% CIs for associations per one standard deviation (SD, calculated in the overall subcohort) of each SFA with incident type 2 diabetes using Prentice-weighted Cox regression,^22^ which allows for over-representation of cases in a case-cohort design, and pooled our findings using random-effects meta-analysis. Heterogeneity between countries was expressed as *I*^2^ values, and we used meta-regression to assess whether the heterogeneity was explained by age, BMI, or sex. We adjusted for potential confounders as follows: model 1 included age (as the underlying timescale), study centre, sex, physical activity index, smoking status, and education level. Model 2 included these parameters plus total energy intake, alcohol intake, and BMI. After recording patterns of association for the nine individual SFAs, we made a post-hoc decision to create three additional exposures based on groupings of SFAs that fit with potential biological action: group 1 (sum of the even-chain SFAs 14:0, 16:0, and 18:0) since these represent both de-novo lipogenesis and dietary intake;^9^, ^11^, ^14^ group 2 (sum of the odd-chain SFAs 15:0 and 17:0) as potential sources of dairy fat;^7^, ^8^ and group 3 (sum of the long- or very-long-chain SFAs 20:0, 22:0, 23:0, and 24:0) since these are under-researched SFAs that might undergo distinct peroxisomal fatty acid metabolism rather than mitochondrial metabolism.^25^ In an additional analysis, we re-grouped 23:0 into group 2 because is it also an odd-chain SFA, and removed it from group 3. Since stearoyl-CoA desaturase-1 catalyses the desaturation of 16:0 to 16:1(n-7) and of 18:0 to 18:1(n-9) through the de-novo lipogenesis pathway, we also estimated stearoyl-CoA desaturase-1 activity using product-to-precursor ratios (ratio of 16:1[n-7] to 16:0 and of 18:1[n-9] to 18:0)^6^, ^15^, ^26^ and assessed each ratio for its association with incident type 2 diabetes.

In a sensitivity analysis based on model 2, we analysed the effects of adjustment for dietary variables (intakes of meat, fruit and vegetables, soft drinks, total dairy products, and carbohydrates [g/day]). We also did an analysis that further accounted for baseline HbA1_C_ value as a covariate. To minimise the possibility of reverse causality, we also excluded 2348 people with HbA1_C_ of 6·5% or higher at baseline or those confirmed as cases of type 2 diabetes (n=1048) within the first 2 years after baseline. Further sensitivity analyses on model 2 included adjustment for: additional potential confounders (dietary carbohydrates intake [g/day] and waist circumference [cm]); comorbidity (prevalent myocardial infarction, stroke, or cancer); and the exclusion of 723 people who were probably dietary misreporters (those with a ratio of energy intake to energy requirement in the bottom or top 1% of the distribution). We also studied the association of SFA quintiles with type 2 diabetes incidence in models 1 and 2.

We postulated that circulating SFAs would be derived from diet and through de-novo lipogenesis, and associated with carbohydrate and alcohol consumption.^9^, ^11^, ^12^ Within the subcohort, we studied associations between each circulating SFA and food intakes, using Pearson correlation coefficients and 95% CI adjusted for age, sex, BMI, and energy intake. We used Stata, version 13.1 for all analyses.

### Role of the funding source

The funders of the study had no role in study design, data collection, data analysis, data interpretation, or writing of the report. The corresponding author had full access to all the data in the study and had final responsibility for the decision to submit for publication.

## Results

In the subcohort participants (mean age 52·3 years [SD 9·2]), SFAs comprised 46% (SD 1·2%) of the total phospholipid fatty acids, with the greatest contributors being the even-chain fatty acids 16:0 (mean 30·1% [SD 1·7%]) and 18:0 (14·1% [1·3%]), whereas 15:0, 17:0, and 20:0–24:0 were present in low relative concentrations (all <0·5%) (table 1). Older adults, those with higher BMI, and men had higher relative concentrations of even-chain SFAs (14:0, 16:0, and 18:0), whereas we noted the opposite for longer-chain SFAs (20:0, 22:0, 23:0, and 24:0) (table 1). Relative concentrations of odd-chain SFAs (15:0 and 17:0) were higher in people with a lower BMI and in women, but were similar across different age groups (table 1). Appendix p 2 shows SFA distribution by country.

**Table 1:** The distribution of plasma phospholipid saturated fatty acids by categories of age, sex, and BMI in the subcohort of the EPIC-InterAct study, and by type 2 diabetes status

	**Distribution of fatty acids (mol%) by subcohort category (n=15 919)**									**Distribution of fatty acids (mol%) by type 2 diabetes status**	
	Age (years)			Sex		BMI (kg/m^2^)*			Total subcohort (n=15 919)	Non-cases (n=15 164)	Type 2 diabetes cases (n=12 132)
	<40 (n=1524)	40–<60 (n=10 919)	≥60 (n=3476)	Men (n=6002)	Women (n=9917)	<25 (n=7052)	25–<30 (n=6255)	≥30 (n=2502)			
All SFAs	45·61 (1·08)	45·94 (1·19)	46·26 (1·25)	46·08 (1·08)	45·91 (1·27)	45·85 (1·22)	46·03 (1·19)	46·21 (1·16)	45·98 (1·21)	45·71 (1·09)	46·13 (1·17)
Myristic acid (14:0)	0·35 (0·11)	0·37 (0·11)	0·39 (0·11)	0·36 (0·10)	0·38 (0·11)	0·38 (0·11)	0·37 (0·11)	0·36 (0·11)	0·37 (0·11)	0·36 (0·11)	0·37 (0·11)
Pentadecanoic acid (15:0)	0·22 (0·06)	0·21 (0·07)	0·22 (0·07)	0·19 (0·07)	0·22 (0·06)	0·23 (0·07)	0·21 (0·07)	0·20 (0·06)	0·21 (0·07)	0·21 (0·06	0·20 (0·06)
Palmitic acid (16:0)	29·88 (1·72)	30·09 (1·69)	30·22 (1·58)	30·32 (1·51)	29·96 (1·75)	30·22 (1·65)	30·09 (1·68)	29·80 (1·67)	30·10 (1·67)	29·93 (1·74)	30·21 (1·67)
Heptadecanoic acid (17:0)	0·42 (0·08)	0·41 (0·09)	0·41 (0·09)	0·39 (0·09)	0·43 (0·09)	0·42 (0·09)	0·41 (0·09)	0·40 (0·10)	0·41 (0·09)	0·41 (0·08)	0·40 (0·09)
Stearic acid (18:0)	13·99 (1·45)	14·11 (1·35)	14·22 (1·24)	14·07 (1·14)	14·151·44)	13·83 (1·31)	14·21 (1·29)	14·72 (1·30)	14·12 (1·34)	14·05 (1·47)	14·22 (1·35)
Arachidic acid (20:0)	0·13 (0·04)	0·13 (0·04)	0·14 (0·04)	0·13 (0·04)	0·13 (0·04)	0·13 (0·04)	0·13 (0·04)	0·13 (0·03)	0·13 (0·04)	0·13 (0·04)	0·13 (0·04)
Behenic acid (22:0)	0·23 (0·08)	0·23 (0·07)	0·24 (0·10)	0·23 (0·08)	0·24 (0·08)	0·23 (0·07)	0·24 (0·09)	0·23 (0·06)	0·24 (0·08)	0·23 (0·07)	0·23 (0·07)
Tricosanoic acid (23:0)	0·10 (0·04)	0·11 (0·05)	0·11 (0·06)	0·10 (0·05)	0·11 (0·05)	0·11 (0·05)	0·11 (0·05)	0·11 (0·04)	0·11 (0·05)	0·10 (0·04)	0·10 (0·05)
Lignoceric (24:0)	0·23 (0·06)	0·23 (0·06)	0·23 (0·08)	0·23 (0·07)	0·23 (0·07)	0·23 (0·06)	0·23 (0·07)	0·22 (0·05)	0·23 (0·07)	0·22 (0·06)	0·22 (0·06)
SFA group 1†	44·23 (1·09)	44·57 (1·21)	44·84 (1·26)	44·76 (1·12)	44·50 (1·27)	44·44 (1·22)	44·66 (1·20)	44·88 (1·20)	44·59 (1·22)	44·34 (1·12)	44·80 (1·20)
SFA group 2‡	0·63 (0·11)	0·62 (0·14)	0·63 (0·14)	0·58 (0·13)	0·65 (0·13)	0·65 (0·13)	0·61 (0·13)	0·60 (0·13)	0·63 (0·13)	0·62 (0·11)	0·60 (0·14)
SFA group 3§	0·70 (0·19)	0·70 (0·18)	0·72 (0·24)	0·69 (0·20)	0·71 (0·20)	0·71 (0·19)	0·70 (0·22)	0·69 (0·16)	0·70 (0·20)	0·69 (0·18)	0·68 (0·19)
Ratio of 16:1 (n-7) to 16:0	0·01 (0·01)	0·02 (0·01)	0·02 (0·01)	0·02 (0·01)	0·02 (0·01)	0·02 (0·01)	0·02 (0·01)	0·02 (0·01)	0·02 (0·01)	0·01 (0·01)	0·02 (0·01)
Ratio of 18:1 (n-9) to 18:0	0·70 (0·15)	0·71 (0·16)	0·70 (0·16)	0·72 (0·16)	0·70 (0·16)	0·73 (0·16)	0·70 (0·16)	0·66 (0·16)	0·71 (0·16)	0·70 (0·15)	0·70 (0·16)

Data are mean (SD). The total subcohort of 15 919 people includes 755 people with incident type 2 diabetes as per the design of a case-cohort study. The 15 164 non-cases represent the subcohort minus the 755 incident cases of type 2 diabetes in this subcohort; this approach enables a comparison of non-cases with cases of type 2 diabetes.

* 110 individuals in the subcohort had missing BMI values, so BMI data are available for only 15 809 people.

† SFA group 1=sum of 14:0, 16:0, and 18:0.

‡ SFA group 2=sum of 15:0 and 17:0.

§ SFA group 3=sum of 20:0, 22:0, 23:0, and 24:0. SFA=saturated fatty acid.

Model 2 in table 2 shows that even-chain SFAs were each positively associated with type 2 diabetes risk individually and when combined (SFA group 1 HR 1·43 [95% CI 1·29–1·58]). By contrast, odd-chain SFAs were each inversely associated with type 2 diabetes individually and when combined (SFA group 2 HR 0·70 [95% CI 0·66–0·74]). Longer-chain SFAs were each inversely associated with type 2 diabetes individually and when combined (SFA group-3 HR 0·70 [95% CI 0·59–0·84]). The results were similar when 23:0 was included in SFA group 2 and removed from group 3 (data not shown). Sensitivity analyses showed the same results (table 2 and appendix p 3). Analyses with quintiles of the fatty acid distributions led to similar conclusions in terms of direction and significance of associations (appendix p 5). For example, results of model 2 showed that the HR (95% CI) for a comparison of quintile 5 versus quintile 1 of even-chain SFAs (SFA group 1) was 3·66 (2·75– 4·87); for odd-chain SFAs (SFA group 2) 0·37 (0·32–0·42); and for longer-chain SFAs (SFA group 3) 0·47 (0·35–0·63); in all cases the p value for the trend was <0·0001.

**Table 2:** Associations between each plasma phospholipid saturated fatty acid, fatty acid groups, and product-to-precursor ratios and type 2 diabetes

	**Model 1, HR (95% CI)**	**Model 2, HR (95% CI)**	**Model 2a, HR (95% CI)**	**Model 2b, HR (95% CI)**	**Model 2c, HR (95% CI)**	**Model 2d, HR (95% CI)**
Myristic acid (14:0)	1·17 (1·11–1·24)	1·15 (1·09–1·22)	1·17 (1·10–1·24)	1·17 (1·11–1·22)	1·13 (1·06–1·21)	1·15 (1·09–1·22)
Pentadecanoic acid (15:0)	0·74 (0·68–0·80)	0·79 (0·73–0·85)	0·79 (0·73–0·86)	0·79 (0·74–0·85)	0·79 (0·74–0·85)	0·80 (0·74–0·86)
Palmitic acid (16:0)	1·22 (1·12–1·33)	1·26 (1·15–1·37)	1·26 (1·15–1·37)	1·19 (1·10–1·28)	1·18 (1·09–1·28)	1·24 (1·13–1·36)
Heptadecanoic acid (17:0)	0·58 (0·54–0·64)	0·67 (0·63–0·71)	0·66 (0·62–0·71)	0·70 (0·67–0·73)	0·70 (0·68–0·73)	0·67 (0·62–0·72)
Stearic acid (18:0)	1·25 (1·17–1·33)	1·06 (1·00–1·13)	1·06 (1·00–1·13)	1·12 (1·08–1·16)	1·12 (1·06–1·18)	1·07 (1·00–1·14)
Arachidic acid (20:0)	0·71 (0·62–0·80)	0·74 (0·65–0·84)	0·74 (0·65–0·84)	0·77 (0·67–0·89)	0·78 (0·68–0·88)	0·76 (0·67–0·86)
Behenic acid (22:0)	0·81 (0·72–0·92)	0·79 (0·69–0·90)	0·79 (0·69–0·90)	0·80 (0·70–0·91)	0·81 (0·71–0·93)	0·80 (0·71–0·91)
Tricosanoic acid (23:0)	0·80 (0·72–0·90)	0·81 (0·72–0·92)	0·81 (0·72–0·92)	0·76 (0·66–0·87)	0·81 (0·72–0·91)	0·83 (0·74–0·93)
Lignoceric acid (24:0)	0·67 (0·57–0·80)	0·72 (0·61–0·85)	0·73 (0·61–0·86)	0·74 (0·63–0·87)	0·75 (0·64–0·88)	0·74 (0·63–0·86)
SFA group 1*	1·55 (1·37–1·76)	1·43 (1·29–1·58)	1·43 (1·29–1·58)	1·39 (1·27–1·52)	1·39 (1·26–1·53)	1·40 (1·27–1·56)
SFA group 2†	0·63 (0·58–0·68)	0·70 (0·66–0·74)	0·69 (0·65–0·74)	0·71 (0·67–0·75)	0·72 (0·69–0·75)	0·70 (0·66–0·75)
SFA group 3‡	0·68 (0·58–0·81)	0·70 (0·59–0·84)	0·71 (0·59–0·84)	0·70 (0·58–0·85)	0·73 (0·62–0·87)	0·73 (0·62–0·86)
Ratio of 16:1 (n-7) to 16:0	1·32 (1·20–1·45)	1·22 (1·13–1·32)	1·22 (1·13–1·32)	1·27 (1·16–1·38)	1·20 (1·12–1·30)	1·22 (1·13–1·32)
Ratio of 18:1 (n-9) to 18:0	0·87 (0·81–0·94)	0·99 (0·93–1·05)	0·98 (0·93–1·04)	0·97 (0·91–1·04)	0·96 (0·91–1·03)	0·99 (0·93–1·05)

Data are pooled HRs (95% CI) per 1 SD difference in plasma phospholipid SFA (12 132 cases of type 2 diabetes and 15 919 people in the subcohort, including 755 cases of type 2 diabetes in the subcohort). HR=hazard ratio. SFA=saturated fatty acid. Model 1: age as the underlying timescale and adjusted for centre, sex, physical activity (inactive, moderately inactive, moderately active, or active), smoking status (never, former, or current), and education level (none, primary school completed, technical or professional school, secondary school, or further education). Model 2: adjusted for factors in model 1 plus total energy intake (continuous, kcal/day), alcohol intake (yes/no), and BMI (continuous, kg/m^2^). Models 2a to 2d show the HR for sensitivity analyses: model 2a: model 2 plus intakes of meat, fruit and vegetables, soft drinks, and total dairy products (continuous, g/day); model 2b: model 2 plus adjustment for baseline HbA1_c_ value (continuous, mmol/mol); model 2c: model 2, repeated after exclusion of 2348 people with HbA1_c_ ≥6·5% (or ≥48 mmol/mol) at baseline; model 2d: model 2, repeated after exclusion of 1048 cases of type 2 diabetes diagnosed within the first 2 years after baseline.

* SFA group 1=sum of 14:0, 16:0, and 18:0.

† SFA group 2=sum of 15:0 and 17:0.

‡ SFA group 3=sum of 20:0, 22:0, 23:0, and 24:0.

**Figure 2:**
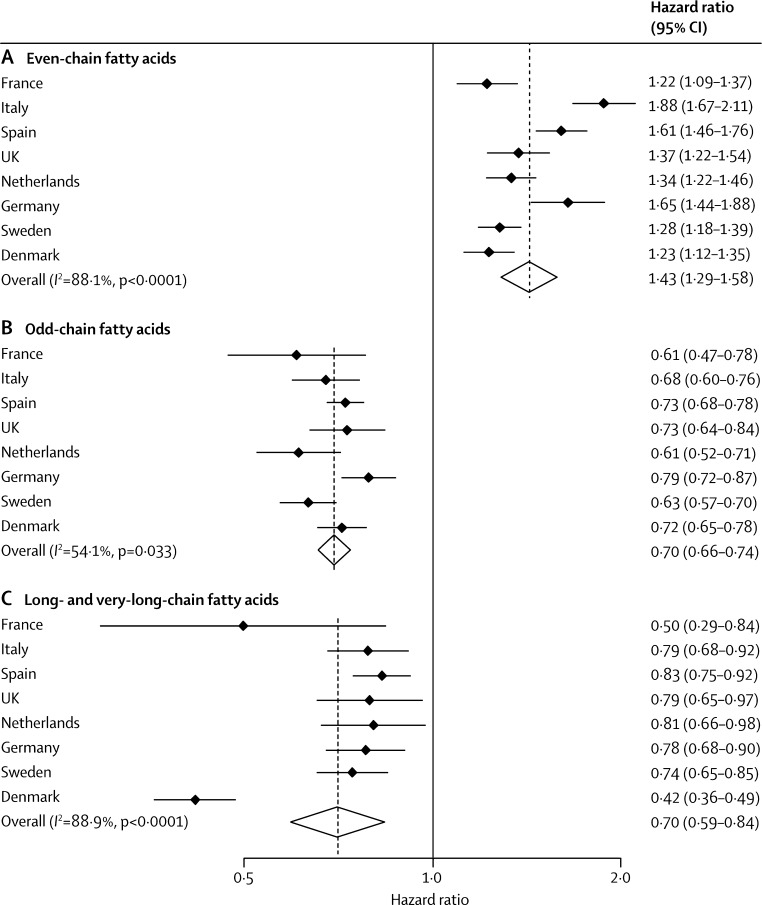
Hazard ratios and 95% CIs for associations between plasma phospholipid saturated fatty acids and incident type 2 diabetes Associations per 1 SD difference in (A) even-chain fatty acids (saturated fatty acid [SFA] group 1: the sum of 14:0, 16:0, and 18:0), (B) odd-chain fatty acids (SFA group 2: the sum of 15:0 and 17:0), and (C) long- and very-long-chain fatty acids (SFA group 3: the sum of 20:0, 22:0, 23:0, and 24:0) and type 2 diabetes. Estimates are per country and the pooled estimate is based on random-effects meta-analysis. The analyses included 12 132 cases of type 2 diabetes and 15 919 people in the subcohort (including 755 individuals with type 2 diabetes in the subcohort); used age as the underlying time variable; and were adjusted for centre, sex, smoking status, alcohol intake, physical activity, education level, total energy intake, and BMI.

Country-specific associations were similar overall (figure 2). Although we recorded evidence of heterogeneity (*I*^2^=88·1%; p<0·0001), meta-regression analyses showed that age, BMI, and sex did not explain this effect (data not shown).

Among estimated markers of stearoyl-CoA desaturase-1 activity, the ratio of 16:1(n-7) to 16:0 was significantly positively associated with type 2 diabetes, but the ratio of 18:1(n-9) to 18:0 was not (table 2; appendix pp 3–5).

**Figure 3:**
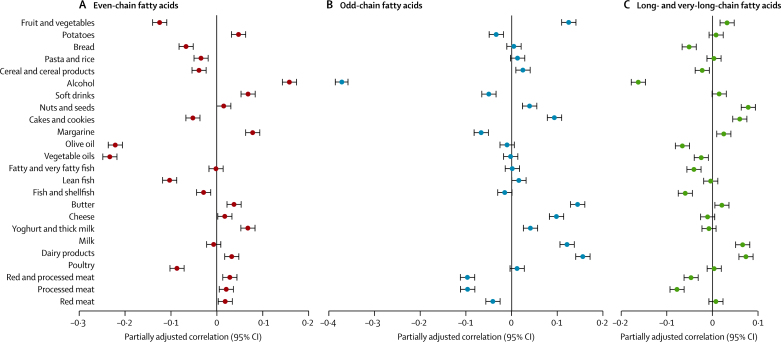
Adjusted Pearson correlation coefficients for the correlation between plasma phospholipid saturated fatty acids (mol%) and types of self-reported food intake (g/day) in the subcohort (n=15 919) Correlations for (A) even-chain fatty acids (saturated fatty acid [SFA] group 1: the sum of 14:0, 16:0 and 18:0), (B) odd-chain fatty acids (SFA group 2: the sum of 15:0 and 17:0), and (C) long- and very-long-chain fatty acids (SFA group 3: the sum of 20:0, 22:0, 23:0 and 24:0). Correlations are adjusted for age, sex, BMI, and total energy intake. Error bars are 95% CIs.

All three groups of SFAs were modestly or weakly correlated with foods, but with overall distinct patterns across fatty acid groups (figure 3). Even-chain SFAs were positively associated with alcohol, soft drinks, margarine, and potatoes, and negatively associated with fruit and vegetables, and both olive oil and vegetable oil (figure 3A). By contrast, odd-chain SFAs generally showed positive associations with dairy products, cakes and cookies, nuts and seeds, and fruit and vegetables, but negative associations with red and processed meat, soft drinks, alcohol, and margarine (figure 3B). The overall correlations for longer-chain SFAs were similar to, but weaker than, those for odd-chain SFAs (figure 3C).

## Discussion

In this large prospective case-cohort study, we investigated the association of nine individual SFAs with the risk of type 2 diabetes, and recorded distinct patterns of association. Even-chain SFAs (14:0, 16:0, and 18:0) were positively associated with the incidence of type 2 diabetes, whereas odd-chain SFAs (15:0 and 17:0) and longer-chain SFAs (20:0, 22:0, 23:0, and 24:0) were inversely associated with type 2 diabetes. Our findings of the differential associations of individual circulating SFAs with the risk of type 2 diabetes are in line with the recent debate about the adverse effects of SFAs.^2^, ^27^ Our results indicate that different SFAs have differential associations with metabolic risk. Therefore, to classify all SFAs as having adverse health effects, as has conventionally been done, does not acknowledge their potentially heterogeneous associations.

The existing evidence around this topic is inconclusive and is based on only a few studies with small sample size, in which the number of participants with type 2 diabetes ranged from 34 in a Finnish study^21^ to 673 in a German study.^15^ Null associations have been recorded for the even-chain SFAs 14:0, 16:0, and 18:0,^17^, ^18^, ^19^, ^20^, ^21^ although positive associations have also been reported for 16:0 and 18:0.^15^, ^16^, ^19^ Some studies reported an inverse association between type 2 diabetes risk and odd-chain SFAs 15:0^17^, ^18^ and 17:0,^16^, ^17^ whereas one showed an inverse association only for the sum of 15:0 and 17:0,^15^ and one a non-significant association with 15:0.^20^ Only one study^15^ reported a significant association with longer-chain SFAs, but showed a positive association for 24:0 (unlike the inverse association recorded in our study). Our findings with 12 132 cases of type 2 diabetes therefore provide the strongest evidence so far for differential associations between nine individual SFAs and type 2 diabetes (panel).

Panel **Research in context**

**Systematic review**

We searched PubMed for relevant articles using terms related to the exposure (“saturated fatty acids” or “saturated fat” or “myristic” or “palmitic” or “stearic” or “myristate” or “palmitate” or “stearate” or “dairy” or “ruminant” or “odd-numbered” or “odd numbered” or “odd chain” or “odd-chain” or “long chain” or “long-chain”), the outcome (“diabetes”), and the prospective study design (“cohort” or “nested”). We reviewed articles resulting from these searches, relevant references cited in those articles, and our own reference databases. Our search showed that past evidence was inconclusive, based on prospective studies with small sample size in which the number of type 2 diabetes events ranged from 34 to 673, and only a small number of individual saturated fatty acids were studied.

**Interpretation**

In the largest study so far, including 12 132 cases of incident type 2 diabetes across eight European countries in whom we measured a set of nine individual plasma phospholipid saturated fatty acids (SFAs), we showed that different individual SFAs were independently associated with incident type 2 diabetes in opposite directions. The inverse associations between diabetes and odd-chain SFAs (15:0 and 17:0)—that are mainly exogenously derived—can be interpreted in terms of dietary dairy fat intake, but the interpretation of positive associations with even-chain SFAs—such as 16:0 and 18:0—is more complex. More research is needed to clarify the extent to which the positive associations between these even-chain SFAs and diabetes reflect complex interplay between intakes of dietary fat, carbohydrates, and alcohol and endogenous de-novo lipogenesis that induces synthesis of even-chain SFAs. Our findings provide strong biological evidence that individual SFAs are not homogenous in their effects, and lend support to the importance of recognising differences between the differential health effects of subtypes of blood SFAs.

Our pattern of findings for the correlations of SFA with food intakes are consistent with previous evidence from feeding intervention studies showing that even-chain SFAs (14:0, 16:0, and 18:0) are derived from de-novo lipogenesis, through which carbohydrates and alcohol are converted to fatty acids in the liver or adipose tissue.^6^, ^9^, ^11^, ^12^ Our finding of a positive association of type 2 diabetes with the product-to-precursor ratio of 16:1(n-7) to 16:0 but not with the 18:1(n-9) to 18:0 ratio (as markers of de-novo lipogenesis) is also consistent with previous observations in smaller samples.^15^, ^17^ However, studies with isotope-labelled dietary fatty acids show that some incorporation of these SFAs into phospholipids occurs,^13^ and evidence also indicates that de-novo lipogenesis is reduced when dietary starch is substituted for sugar, which suggests the importance of carbohydrate type.^11^ The extent to which de-novo lipogenesis might also contribute to the circulating levels of these SFAs could be quite low under conditions of relatively high-fat habitual diets in free-living Western populations.^14^ Because existing knowledge is insufficient, future research is needed to quantify the effects of habitual diets and metabolic state on blood concentrations of SFAs. Importantly, measurements of even-chain SFAs in the blood should not be used to directly interpret dietary SFAs.

Given that de-novo lipogenesis is the underlying process for increased levels of even-chain SFAs, this pathway might increase the risk of type 2 diabetes through hepatic steatosis and related mechanisms.^28^ Direct effects of even-chain SFAs could also promote the development of type 2 diabetes: biochemical studies indicate toxic effects specifically of 16:0, including activation of inflammatory cytokines and lipotoxicity to pancreatic β cells.^29^ Epidemiological research cannot address biological mechanisms and should therefore be complemented by experimental studies.

Our finding of positive correlations of dairy products with odd-chain SFAs (15:0 and 17:0) is consistent with previous evidence that these SFAs are markers of exogenous origin dairy fat intake.^6^, ^7^, ^8^ Although residual confounding by other dairy components such as vitamin D or calcium cannot be ruled out, and nor can the possible effects of processes related to fermentation of dairy products,^4^, ^5^, ^30^ our strong findings for odd-chain SFAs in the blood lend support to accumulating evidence of an inverse association between dairy products and type 2 diabetes risk.^4^, ^5^

Our report of an inverse association between the very-long-chain SFAs and type 2 diabetes provides the largest appraisal so far of an as-yet under-researched group of SFAs that might undergo distinct fatty acid metabolism through peroxisomal fatty acid oxidation.^25^ To our knowledge, the sources and metabolic effects of very-long-chain SFAs are largely unknown, and our new findings should provide the impetus for further research into this group of fatty acids.

The main limitation of our study was that SFAs were measured at one timepoint only, and intra-individual variation over time is likely. However, we would not expect any errors to be differential with respect to case status. We could only assess relative—not absolute—concentrations of fatty acids (in mol%), but this valid approach is often used in epidemiological research and tends to provide a better interpretation of metabolic inter-relationships than do absolute measurements.^6^ Fatty acids were measured in long-term stored samples, but stability is likely given that samples were stored at −196°C.^6^ Plasma phospholipid SFAs might be indicative of both dietary and metabolic influences, affected by interplay of complex exogenous and genetic factors, which we were unable to tease out. This raises caution against inferring dietary consumption on the basis of measurements of SFAs that are not exclusively exogenously derived. Our indirect estimation of fatty acid desaturation ratios has limited use as a biomarker of stearoyl-CoA desaturase-1 activity,^26^ but direct measures involving liver biopsies or tracer techniques are not feasible in epidemiological studies. Our outcome ascertainment was complete for the entire cohort and therefore does not have the problem of ascertainment bias consequent on a requirement for attendance at a follow-up visit, but was limited by a reliance on clinically incident diabetes. However, we minimised false positives by applying rigorous verification criteria to ensure that no case was included unless verified by at least two independent sources. Although false negatives were also possible because of undiagnosed incident diabetes, such misclassification can be assumed to be non-differential with regard to the exposure and therefore any potential bias would be unlikely to change our conclusions based on a relative scale (HR) in our analysis. We also minimised the potential for reverse causation bias from undiagnosed prevalent diabetes by applying three separate sensitivity analyses to test the robustness of our results: adjustment for baseline HbA1_C_, exclusion of those with raised HbA1_C_ at baseline, and exclusion of those with diabetes diagnosed within 2 years of baseline.

The strengths of our study include the large sample size, prospective study design and hence the ability to study the temporality of association with type 2 diabetes, long follow-up, the inclusion of populations from eight European countries with diverse dietary intakes, comprehensive type 2 diabetes case ascertainment and verification, adjustment for many potential confounders, and a series of sensitivity analyses. We were able to analyse the associations of type 2 diabetes with a large number of objectively measured SFAs.

In conclusion, our findings indicate that different individual plasma phospholipid SFAs are differentially associated with risk of type 2 diabetes, which supports the importance of recognising that individual blood SFAs exert heterogeneous effects. Further research into an increased understanding of dietary versus endogenous metabolic sources of circulating individual SFAs will help to inform updated public health messages about the dietary intake of saturated fats and their sources.
