# Supplementary material for: Differences in the prospective association between individual plasma phospholipid saturated fatty acids and incident type 2 diabetes: the EPIC-InterAct case-cohort study
Source: Lancet Diabetes Endocrinol. 2014 Oct;2(10):810–8. doi: 10.1016/S2213-8587(14)70146-9 (PMC4196248; doi:10.1016/S2213-8587(14)70146-9)
Supplement: Supplementary appendix [file mmc1.pdf]

THE LANCET Diabetes & Endocrinology

Supplementary appendix

This appendix formed part of the original submission and has been peer reviewed. We post it as supplied by the authors.

Supplement to: Forouhi NG, Koulman A, Sharp SJ, et al. Differences in the prospective association between individual plasma phospholipid saturated fatty acids and incident type 2 diabetes: the EPIC-InterAct case-cohort study. *Lancet Diabetes Endocrinol* 2014; published online Aug 6. http://dx.doi.org/10.1016/S2213-8587(14)70146-9.

Online appendix materials

Appendix Table 1: The distribution of individual saturated fatty acids and groups of fatty acids in the study subcohort by country - EPIC-InterAct study

Appendix Table 2: Sensitivity analyses for the associations between each saturated fatty acid, fatty acid groups and product-to-precursor ratios and type 2 diabetes: EPIC-InterAct Study

Appendix Table 3: Pooled hazard ratio (HR) and 95% CI for associations between quintiles of each saturated fatty acid, fatty acid groups and product-to-precursor ratios and type 2 diabetes: EPIC-InterAct Study

Appendix Table 1: The distribution of individual saturated fatty acids and groups of fatty acids in the study subcohort by country - EPIC-InterAct study

**Fatty acids**	**France N=587**		**Italy N=2006**		**Spain N=3555**		**UK N=1310**		**Netherlands N=1500**		**Germany N=2045**		**Sweden N=2826**		**Denmark N=2090**		**Total N=15919**	
	**mean**	**sd**	**mean**	**sd**	**mean**	**sd**	**mean**	**sd**	**mean**	**sd**	**mean**	**sd**	**mean**	**sd**	**mean**	**sd**	**mean**	**sd**
Total saturated fatty acids	47·0	1·8	45·7	1·1	45·5	1·1	46·3	1·5	46·2	1·4	45·8	0·9	46·3	1·1	46·2	1·0	46·0	1·2
Myristic acid (14:0)	0·43	0·12	0·37	0·09	0·29	0·08	0·39	0·11	0·41	0·11	0·40	0·11	0·41	0·10	0·40	0·10	0·37	0·11
Pentadecanoic acid (15:0)	0·27	0·07	0·21	0·05	0·18	0·05	0·24	0·08	0·25	0·08	0·22	0·07	0·21	0·05	0·21	0·05	0·21	0·07
Palmitic acid (16:0)	30·5	1·6	30·0	1·4	29·0	1·6	30·3	1·6	30·2	1·8	30·3	1·5	30·6	1·5	30·9	1·5	30·1	1·7
Heptadecanoic acid (17:0)	0·50	0·11	0·41	0·07	0·44	0·10	0·42	0·09	0·41	0·09	0·39	0·07	0·40	0·08	0·38	0·08	0·41	0·09
Stearic acid (18:0)	14·4	1·5	14·0	1·2	14·8	1·2	14·0	1·4	14·0	1·6	13·8	1·3	14·0	1·1	13·7	1·2	14·1	1·3
Arachidic acid (20:0)	0·14	0·03	0·13	0·03	0·13	0·03	0·16	0·05	0·14	0·05	0·13	0·03	0·13	0·03	0·12	0·03	0·13	0·04
Behenic acid (22:0)	0·25	0·06	0·22	0·07	0·23	0·06	0·27	0·10	0·29	0·14	0·21	0·07	0·24	0·06	0·22	0·05	0·24	0·08
Tricosanoic acid (23:0)	0·13	0·05	0·11	0·04	0·10	0·03	0·13	0·07	0·13	0·07	0·09	0·05	0·11	0·03	0·10	0·04	0·11	0·05
Lignoceric acid (24:0)	0·26	0·06	0·23	0·06	0·23	0·05	0·24	0·08	0·28	0·11	0·21	0·06	0·22	0·05	0·21	0·04	0·23	0·07

Appendix Table 2: Sensitivity analyses for the associations between each saturated fatty acid, fatty acid groups and product-to-precursor ratios and type 2 diabetes: EPIC-InterAct Study

**Saturated fatty acid**	**Model 2**	**Model 2A**	**Model 2B**	**Model 2C**	**Model 2D**
Myristic (14:0)	1·15 (1·09, 1·22)	1·16 (1·10, 1·23)	1·14 (1·07, 1·21)	1·16 (1·10, 1·22)	1·15 (1·09· 1·22)
Pentadecanoic (15:0)	0·79 (0·73, 0·85)	0·80 (0·73, 0·86)	0·81 (0·75, 0·87)	0·79 (0·73, 0·86)	0·79 (0·73, 0·85)
Palmitic (16:0)	1·26 (1·15, 1·37)	1·26 (1·15, 1·37)	1·22 (1·12, 1·34)	1·26 (1·16, 1·37)	1·26 (1·15, 1·37)
Heptadecanoic (17:0)	0·67 (0·63, 0·71)	0·67 (0·63, 0·70)	0·71 (0·67, 0·75)	0·67 (0·63, 0·71)	0·67 (0·63, 0·71)
Stearic (18:0)	1·06 (1·00, 1·13)	1·06 (1·00, 1·13)	1·05 (0·99, 1·11)	1·06 (1·00, 1·12)	1·06 (1·00, 1·13)
Arachidic (20:0)	0·74 (0·65, 0·84)	0·74 (0·64, 0·84)	0·74 (0·66, 0·84)	0·74 (0·65, 0·84)	0·74 (0·65, 0·84)
Behenic (22:0)	0·79 (0·69, 0·90)	0·78 (0·68, 0·90)	0·82 (0·72, 0·93)	0·79 (0·69, 0·90)	0·79 (0·69, 0·90)
Tricosanoic (23:0)	0·81 (0·72, 0·92)	0·81 (0·72, 0·92)	0·82 (0·72, 0·93)	0·82 (0·72, 0·92)	0·81 (0·72, 0·92)
Lignoceric (24:0)	0·72 (0·61, 0·85)	0·72 (0·61, 0·86)	0·75 (0·64, 0·88)	0·72 (0·61, 0·86)	0·72 (0·61, 0·85)
SFA group 1	1·43 (1·29, 1·58)	1·42 (1·28, 1·58)	1·37 (1·24, 1·53)	1·43 (1·31, 1·55)	1·43 (1·29, 1·58)
SFA group 2	0·70 (0·66, 0·74)	0·70 (0·65, 0·74)	0·73 (0·69, 0·77)	0·70 (0·66, 0·74)	0·70 (0·66, 0·74)
SFA group 3	0·70 (0·59, 0·84)	0·70 (0·58, 0·84)	0·74 (0·62, 0·87)	0·70 (0·58, 0·84)	0·70 (0·59, 0·84)
16:1n7 to 16:0 ratio	1·22 (1·13, 1·32)	1·23 (1·14, 1·33)	1·17 (1·10, 1·25)	1·22 (1·13, 1·32)	1·22 (1·13, 1·32)
18:1n9 to 18:0 ratio	0·99 (0·93, 1·05)	0·99 (0·93, 1·05)	0·98 (0·93, 1·03)	0·99 (0·94, 1·05)	0·99 (0·93, 1·05)

Pooled hazard ratios and 95% confidence intervals are per 1 SD difference in plasma phospholipid SFA (12,132 T2D cases and 15,919 sub-cohort, including 755 T2D cases in sub-cohort).

Model 2 is the 2 is the model adjusted for age as underlying timescale, centre, sex, physical activity (inactive, moderately inactive, moderately active, active), smoking (never, former, current), education (none, primary school completed, technical or professional school, secondary school, longer education), total energy intake (continuous, kcal/day), alcohol intake (yes/no), BMI (continuous, kg/m^2).
Models 2A to2C show the HR for sensitivity analyses:
Model A: additionally adjusted for carbohydrates intake (continuous, g/d)
Model B: additionally adjusted for waist circumference (continuous, cm);
Model C: additionally adjusted for prevalent cardiovascular disease (myocardial infarction or stroke) and prevalent cancer (all yes/no);
Model D: with exclusion of 723 individuals who were likely to be energy mis-reporters.

SFA group 1: sum of 14:0, 16:0 and 18:0
SFA group 2: sum of 15:0 and 17:0
SFA group 3: sum of 20:0, 22:0, 23:0 and 24:0

Appendix Table 3: Pooled hazard ratio (HR) and 95% CI for associations between quintiles of each saturated fatty acid, fatty acid groups and product-to-precursor ratios and type 2 diabetes: EPIC-InterAct Study

	Q2 vs Q1			Q3 vs Q1			Q4 vs Q1			Q5 vs Q1			Per quintile			*p* value for
	**HR**	**Lower**	**Upper**	**HR**	**Lower**	**Upper**	**HR**	**Lower**	**Upper**	**HR**	**Lower**	**Upper**	**HR**	**Lower**	**Upper**	**trend**
Model 1																
Myristic acid (14:0)	1·30	1·16	1·45	1·53	1·38	1·69	1·74	1·53	1·99	1·64	1·47	1·83	1·14	1·11	1·18	<0·0001
Pentadecanoic acid (15:0)	0·86	0·79	0·93	0·74	0·68	0·81	0·62	0·54	0·71	0·46	0·37	0·56	0·82	0·78	0·86	<0·0001
Palmitic acid (16:0)	1·19	1·05	1·34	1·39	1·16	1·66	1·58	1·26	1·97	1·75	1·35	2·27	1·16	1·09	1·23	<0·0001
Heptadecanoic acid (17:0)	0·65	0·57	0·75	0·41	0·33	0·51	0·30	0·24	0·37	0·24	0·20	0·30	0·68	0·64	0·71	<0·0001
Stearic acid (18:0)	1·04	0·96	1·14	1·15	0·99	1·33	1·47	1·23	1·75	1·75	1·46	2·09	1·16	1·12	1·21	<0·0001
Arachidic acid (20:0)	0·65	0·56	0·76	0·55	0·47	0·64	0·48	0·40	0·56	0·42	0·33	0·53	0·80	0·76	0·85	<0·0001
Behenic acid (22:0)	0·81	0·73	0·90	0·69	0·58	0·82	0·62	0·50	0·76	0·59	0·46	0·77	0·87	0·81	0·93	<0·0001
Tricosanoic acid (23:0)	0·82	0·67	1·01	0·66	0·54	0·82	0·67	0·53	0·84	0·56	0·48	0·66	0·86	0·82	0·90	<0·0001
Lignoceric acid (24:0)	0·63	0·55	0·72	0·56	0·46	0·68	0·49	0·41	0·59	0·42	0·34	0·51	0·80	0·75	0·85	<0·0001
Cis-16:1 to cis-16:0 ratio	1·46	1·32	1·61	1·85	1·67	2·05	2·50	2·25	2·78	3·37	2·82	4·03	1·35	1·30	1·41	<0·0001
Cis-18:1n9c to cis-18:0 ratio	0·85	0·78	0·92	0·83	0·74	0·93	0·73	0·63	0·83	0·68	0·55	0·86	0·91	0·87	0·96	<0·0001
SFA group 1	1·75	1·44	2·13	2·66	2·07	3·41	4·07	2·99	5·54	5·63	4·24	7·47	1·52	1·43	1·62	<0·0001
SFA group 2	0·70	0·62	0·79	0·50	0·44	0·56	0·37	0·31	0·44	0·27	0·23	0·32	0·72	0·68	0·75	<0·0001
SFA group 3	0·71	0·61	0·81	0·52	0·43	0·64	0·48	0·39	0·59	0·45	0·37	0·55	0·80	0·75	0·85	<0·0001
Model 2																
Myristic acid (14:0)	1·22	1·07	1·38	1·35	1·18	1·54	1·51	1·29	1·77	1·48	1·26	1·73	1·12	1·08	1·17	<0·0001
Pentadecanoic acid (15:0)	0·91	0·79	1·04	0·77	0·69	0·86	0·67	0·58	0·77	0·53	0·44	0·64	0·85	0·82	0·89	<0·0001
Palmitic acid (16:0)	1·21	1·03	1·43	1·50	1·21	1·85	1·68	1·30	2·16	1·91	1·43	2·56	1·19	1·11	1·27	<0·0001
Heptadecanoic acid (17:0)	0·74	0·68	0·82	0·51	0·45	0·57	0·39	0·34	0·46	0·34	0·28	0·42	0·74	0·71	0·77	<0·0001
Stearic acid (18:0)	0·96	0·85	1·09	0·95	0·83	1·08	1·15	0·99	1·33	1·12	0·95	1·33	1·04	1·01	1·08	0·009
Arachidic acid (20:0)	0·71	0·60	0·86	0·60	0·48	0·73	0·50	0·41	0·62	0·48	0·35	0·65	0·82	0·76	0·88	<0·0001
Behenic acid (22:0)	0·78	0·69	0·87	0·69	0·58	0·82	0·59	0·47	0·72	0·56	0·43	0·74	0·85	0·80	0·91	<0·0001
Tricosanoic acid (23:0)	0·75	0·64	0·88	0·65	0·52	0·82	0·66	0·53	0·83	0·54	0·43	0·67	0·86	0·81	0·91	<0·0001
Lignoceric acid (24:0)	0·64	0·54	0·76	0·60	0·47	0·76	0·56	0·47	0·67	0·47	0·37	0·60	0·83	0·78	0·89	<0·0001
Cis-16:1 to cis-16:0 ratio	1·41	1·26	1·57	1·66	1·48	1·86	2·06	1·80	2·36	2·35	2·02	2·74	1·24	1·19	1·29	<0·0001
Cis-18:1n9c to cis-18:0 ratio	0·94	0·83	1·05	0·92	0·83	1·02	0·89	0·78	1·02	0·92	0·77	1·09	0·98	0·94	1·02	0·333
SFA group 1	1·50	1·28	1·76	2·00	1·62	2·48	2·94	2·26	3·84	3·66	2·75	4·87	1·38	1·29	1·48	<0·0001
SFA group 2	0·75	0·68	0·82	0·57	0·52	0·63	0·44	0·38	0·51	0·37	0·32	0·42	0·77	0·74	0·79	<0·0001
SFA group 3	0·73	0·60	0·89	0·54	0·43	0·69	0·51	0·40	0·65	0·47	0·35	0·63	0·81	0·75	0·87	<0·0001

Hazard Ratios (HRs) compare quintiles (Q) Q2-Q5 with Q1, where Q1-Q5 were defined by values which divide the distribution of the fatty acid in the overall sub-cohort into fifths.

Model 1: Age as underlying time variable, and adjusted for centre, sex, physical activity (inactive, moderately inactive, moderately active, active), smoking (never, former, current), education level (none, primary school completed, technical or professional school, secondary school, longer education)..

Model 2: Adjusted as in model 1 + total energy intake (continuous, kcal/day), alcohol (yes/no), BMI (continuous, kg/m^2).

SFA group 1: sum of 14:0, 16:0 and 18:0

SFA group 2: sum of 15:0 and 17:0

SFA group 3: sum of 20:0, 22:0, 23:0 and 24:0
